# The Impact of the COVID-19 Pandemic on the Psychological Well-Being of Caregivers of People with Dementia or Mild Cognitive Impairment: A Systematic Review and Meta-Analysis

**DOI:** 10.3390/geriatrics8050097

**Published:** 2023-09-28

**Authors:** Pinar Soysal, Nicola Veronese, Lee Smith, Yaohua Chen, Burcu Akpinar Soylemez, Alessandra Coin, Dorota Religa, Tarja Välimäki, Mariana Alves, Susan D. Shenkin

**Affiliations:** 1 Department of Geriatric Medicine, Faculty of Medicine, Bezmialem Vakif University, Istanbul 34093, Turkey; 2 Department of Internal Medicine, Geriatrics Section, University of Palermo, 90133 Palermo, Italy; 3 Centre for Health Performance and Wellbeing, Anglia Ruskin University, Cambridge CB1 1PT, UK; 4 Department of Geriatrics, Lille Neurosciences & Cognition, University of Lille, 59000 Lille, France; 5 Department of Internal Medicine Nursing, Faculty of Nursing, Dokuz Eylul University, Izmir 35210, Turkey; 6 Geriatrics Division, Department of Medicine, University of Padua, 35122 Padua, Italy; 7 Division for Clinical Geriatrics, Department of Neurobiology, Care Sciences and Society (NVS), Karolinska Institute, 14152 Stockholm, Sweden; 8 Department of Nursing Science, University of Eastern Finland, 70211 Kuopio, Finland; 9 Serviço de Medicina III, Hospital Pulido Valente, CHLN, Alameda das Linhas de Torres, 117, 1769-001 Lisbon, Portugal; 10 Ageing and Health Research Group, Usher Institute, University of Edinburgh, Edinburgh EH16 4SB, UK

**Keywords:** COVID-19, psychological well-being, caregivers, dementia, systematic review, meta-analysis

## Abstract

The aim of this systematic review was to investigate the effects of the COVID-19 lockdown on the psychological well-being of caregivers of people with dementia or mild cognitive impairment (PwD/MCI). Electronic databases were searched from inception to August 2022 for observational studies investigating the COVID-19 lockdown and psychological well-being of caregivers of PwD/MCI. Summary estimates of standardized mean differences (SMD) in psychological well-being scores pre- versus during COVID-19 were calculated using a random-effects model. Fifteen studies including 1702 caregivers (65.7% female, mean age 60.40 ± 12.9 years) with PwD/MCI were evaluated. Five studies found no change in psychological well-being parameters, including depression, anxiety, distress, caregiver burden, and quality of life. Ten studies found a worsening in at least one parameter: depression (six studies, *n* = 1368; SMD = 0.40; 95%CI: 0.09–0.71; *p* = 0.01, *I*^2^ = 86.8%), anxiety (seven studies, *n* = 1569; SMD = 1.35; 95%CI: 0.05–2.65; *I*^2^ = 99.2%), caregiver distress (six studies, *n* = 1320, SMD = 3.190; 95%CI: 1.42–4.95; *p* < 0.0001; *I*^2^ = 99.4%), and caregiver burden (four studies, *n* = 852, SMD = 0.34; 95%CI: 0.13–0.56; *p* = 0.001; *I*^2^ = 54.1%) (*p* < 0.05). There was an increase in depression, anxiety, caregiver burden, and distress in caregivers of PwD/MCI during the lockdown in the COVID pandemic. This could have longer term consequences, and it is essential that caregivers’ psychological well-being is assessed and supported, to benefit both themselves and those for whom they care.

## 1. Introduction

The COVID-19 pandemic has influenced many conditions related to neurodegenerative disease both during the pandemic and beyond. For example, survivors of COVID-19 infection had a higher incidence of new-onset Alzheimer’s Disease (AD) and dementia (especially older people) compared to those without COVID-19 and other respiratory infections [1]. Also, COVID-19 was likely a significant contributor to the large increase in deaths from dementia in recent years [2]. Importantly, the COVID-19 pandemic has posed significant social, psychological, emotional, and physical challenges to family members and friends who provide care for people with dementia, thereby creating a crisis for caregivers [3]. Approximately 30% of older adults with dementia rely on three or more unpaid caregivers, compared to 23% of older adults without dementia [3]. Therefore, the COVID-19 pandemic per se and its wider psychosocial impact may have affected multiple caregivers.

Efforts to stop the spread of SARS-CoV-2 led to restrictions on in-person contact, including “stay-at-home” recommendations to avoid infection, simultaneously with health service interruptions [4]. Due to the “stay-at-home” recommendations, caregivers endured disrupted daily routines, fewer supportive services for themselves and their care recipient, and reduced social relations [5]. The consequences of the pandemic have been noteworthy in vulnerable populations, increasing existing health inequalities [6]. These consequences will likely build on existing social inequalities and disproportionately affect vulnerable populations, such as caregivers.

Lockdown restrictions were associated with worsening neuropsychiatric symptoms in people living with dementia and those with mild cognitive impairment (MCI) [7,8,9]. There was a significant emotional burden for caregivers [7,8]. In a nationwide survey in Italy during the lockdown in 2020, caregivers (*n* = 5321) reported a significant increase in anxiety (45.9%), depression (18.6%), irritability (26.2%), and distress (28.9%) during quarantine [10]. Notably, in the one-year follow-up of Italian caregivers (*n* = 85), stress-related symptoms stayed high, with depressive symptoms and feelings of sadness being the most prevalent [11].

The COVID-19 pandemic has posed significant social, psychological, emotional, and physical challenges to caregivers, but detail is lacking on the specific consequences including depression, anxiety, distress, caregiver burden, mental well-being, and quality of life. Importantly, there is currently no meta-analysis investigating how caregivers’ general psychological well-being was affected by the COVID-19 pandemic. Therefore, the aim of this review is to examine the effect of the COVID-19 pandemic on caregivers of people with dementia (PwD) or MCI.

## 2. Method

This systematic review was conducted according to the Strengthening the Reporting of Observational Studies in Epidemiology (STROBE) criteria [12] and reported according to the Preferred Reporting Items for Systematic Reviews and Meta-Analyses (PRISMA) statement [13]. The present review was pre-registered with PROSPERO [registration number: CRD42022349890].

### 2.1. Search Strategy

We searched MEDLINE/PubMed (via Ovid), Embase, Scopus, CINAHL (Cumulative Index to Nursing and Allied Health Literature), PsycINFO, and Web of Science. We built detailed and highly sensitive search strategies, combining search terms (free vocabulary words and controlled vocabulary terms) for dementia, COVID-19, and caregivers with the help of an expert librarian, and searched from database inception to 7 August 2022.

The search terms used were (dement * OR Alzheimer * OR Lewy OR Posterior cortical atrophy OR Binswanger OR Progressive supranuclear palsy OR Frontotemporal disorder * OR Frontotemporal degeneration OR Corticobasal degeneration OR Corticobasal syndrome OR Mild cognitive impairment) AND (Caregiver OR Carer OR Caring OR family OR Relative OR Spouse OR Children) AND (COVID-19 OR Novel Coronavirus–Infected Pneumonia OR 2019 novel coronavirus OR 2019-nCoV OR SARS-CoV-2) ti,ab.

### 2.2. Inclusion and Exclusion Criteria

The included studies were as follows: (i) studies conducted during the COVID-19 pandemic, (ii) longitudinal, cohort, or case–control observational studies, (iii) studies that involved unpaid/informal caregivers (e.g., family members) or paid caregivers, (iv) studies that involved caregivers for people with a prior diagnosis of dementia or MCI (or another condition defined as cognitive decline which has an increased risk of future dementia), and (v) studies that reported caregiver burden using validated scales [secondary outcomes depression, anxiety, and distress using validated scales].

Studies were excluded if they were as follows: (i) qualitative or thematic studies, (ii) conference abstracts, (iii) cross-sectional studies, (iv) studies that were carried out prior to and not including data during the COVID-19 pandemic, (v) studies that were not longitudinal in design, (vi) studies that included caregivers for persons without MCI/dementia, and (viii) studies that did not assess outcomes using validated scales.

### 2.3. Data Extraction and Statistical Analyses

The literature search, assessment of inclusion and exclusion criteria, quality of studies, and extraction of data were independently undertaken and verified by two authors (DR, AC). The results were then compared, and, in case of inconsistency, consensus was reached with the participation of a third author (PS). The following information was extracted: (1) characteristics of the study population (e.g., sample size, demographics, country in which the study was performed, and date of data collection); (2) setting in which the study was performed (i.e., own home or residential facility); (3) definition of caregivers (paid, unpaid, family member or not, and living with the PwD/MCI or not); (4) presence of dementia/MCI, and dementia type; and severity of cognitive impairment (MMSE, CDR); (5) pre-COVID-19 (or early in lockdown) and later in lockdown caregivers’ mental health outcomes (caregiver burden, anxiety, distress, depression). measured using validated scales (e.g., GAD7, PHQ9).

### 2.4. Meta-Analysis Method

We synthesized the results using a meta-analysis when at least three studies for the same outcome were present. The data were reported as standardized mean differences (SMDs) with 95% confidence intervals (CIs), under a random-effects model.

In case of significant heterogeneity (identified as *I*^2^ ≥ 50%), we planned to run meta-regression analyses, but since less than ten studies were present for each outcome, this analysis was not performed. Similarly, sensitivity analyses planned by protocol were not carried out since less than three studies were present for each stratum. Publication bias was assessed using Egger’s test and, in case of a publication bias, a trim-and-fill analysis was planned.

For all analyses, a *p*-value less than 0.05 was considered statistically significant. All analyses were performed using STATA version 14.0 (StataCorp, College Station, TX, USA).

### 2.5. Assessment of Study Quality/Risk of Bias

The Risk of Bias in Non-randomized Studies—of Exposure (ROBINS-E) tool was used to assess the risk of bias of the selected studies. ROBINS-E provides a structured approach to assessing the risk of bias in the observational epidemiological studies [14]. ROBINS-E was used to provide a thorough examination of the strength of evidence about the following categories: Domain 1: Risk of bias due to confounding • Domain 2: Risk of bias arising from measurement of the exposure • Domain 3: Risk of bias in selection of participants into the study (or into the analysis) • Domain 4: Risk of bias due to post-exposure interventions • Domain 5: Risk of bias due to missing data • Domain 6: Risk of bias arising from measurement of the outcome • Domain 7: Risk of bias in selection of the reported result. The risk of bias was recorded as either low risk of bias, some concerns, high risk of bias, or very high risk of bias. Two researchers independently assessed all items (YC, BA), and disagreements were resolved by consensus in consultation with a third researcher (PS or SDS).

## 3. Results

### 3.1. Search Results

**Figure 1:**
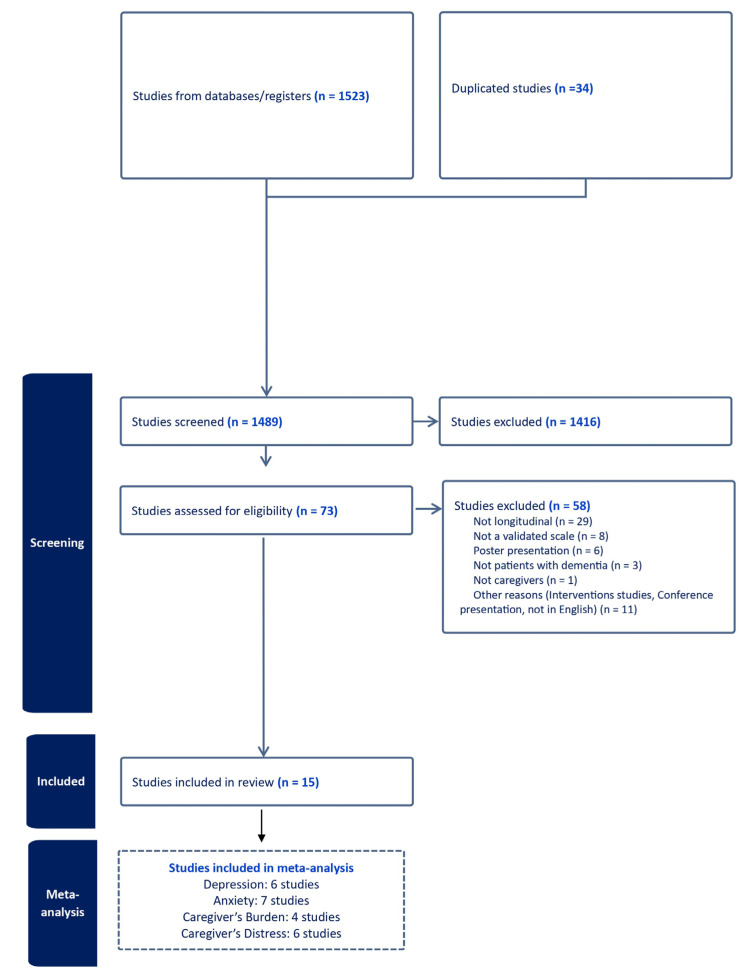
PRISMA flow-chart.

Of 1489 studies, we examined 73 articles as full texts (Figure 1), excluding 58 articles, mainly due to data being cross-sectional. A total of 15 studies were included in the review [11,15,16,17,18,19,20,21,22,23,24,25,26,27,28].

### 3.2. Descriptive Results (Table 1)

The majority of the studies (*n* = 12) were conducted in Europe [11,15,17,18,19,21,22,23,24,25,26,28], and three in Asia [16,20,27]. A total of 1702 caregivers of PwD/MCI were included (mean age 60.40 ± 12.9 years, 65.7% female). PwD were older, with a mean age of 76.36 ±9.32 years. Caregivers were more likely to be female than PwD (65.7% vs. 52.0%). Almost all caregivers were unpaid (>90%); 52.2% were spouses and were living with PwD together (72.9%).

**Table 1:** Descriptive data of the included studies.

Author (s), Year	Country	CG/ PwD, *n*	Mean Age	Female, *n*	Characteristics of CG *n*	Mean Age	Female, *n*	Dementia Types	Dementia Severity	Caregiver Measurement	Lockdown Duration, Weeks	General Findings	RoB
Altieri et al., 2021 * DA [15]	Italy	84	48.7 (11.7)	71 (84.5%)	Living with PwD (63) Unpaid (84) Family (84) -Spouses (10) 11.9%	78.5 (10.1)	61 (72.6%)	AD (47) VAD (26) FTD (9) LBD (2)	Severe (29) Moderate (38) Mild (17)	HADS scale CBI RSA	8 weeks	- Increase in depression level - High resilience had a negative effect on anxiety	High
Bao et al., 2022 * CB [16]	China	177	62.5 (10.8)	78 (44.1%)	Living with PwD (130) Unpaid (177) Family (177) -Spouses (102) 11.9%	71.0 (8.2)	100 (56.5%)	AD (105) DLB (22) MCI (50)	NA	GAD-7 PHQ-9 PSQI ZBI	52 weeks	- Increased caregiver burden and worsened psychological states	Some
Borges-Machado et al., 2020 * CB [17]	Portugal	36	64.94 (13.5)	15 (41.7%)	NA Unpaid (36) Family (36) -Spouses (22) 61.1%	74.28 (6.76)	24 (66.7%)	AD (17) VAD (2) FTD (1) MCI (5) Others(11)	NA	CarerQol-7D CarerQol-VAS	12 weeks	- Increased caregiving burden and decline in well-being.	Very high
Bussè et al., 2022 * DACB [11]	Italy	85	62 (14.6)	59 (69.4%)	Living with PwD (57) Unpaid (85) Family (85) -Spouses (49) 57.6%	74.62 (11.3)	NA	AD (51) DLB (26) FTD (6) VAD (2)	Severe (18) Moderate (16) Mild (51)	CBI PSQI DASS-21	52 weeks	- Caregiver burden was higher during pandemic and time dependent - Caregivers reported at least one stress-related symptom (depression, irritability, anxiety, and sleep alterations)	Some
Carbone et al., 2021 [18]	Italy	35	61.23 (9.91)	26 (74.28%)	NA Unpaid (34) Family(34) -Spouses (NA)	82.60 (8.91)	22 (62.9%)	AD (6) VAD (13) Others(16)	Severe (13) Moderate and Mild (22)	NPI caregiver distress score	8 weeks	No change was found in caregiver’s distress	High
Daley et al., 2022 [19]	United Kingdom	248	70.08 (10.6)	169 (68.1%)	Living with PwD (157) Unpaid (248) Family(248) -Spouses (197) 79.4%	77.47 (8.0)	103 (41.5%)	AD (93) VAD (37) Others (118)	Severe (11) Moderate (73) Mild (156)	C-DEMQOL	T1-T2: 61 weeks T2-T3: 20 weeks	No change was found in caregiver’s quality of life	Some
Fong at al., 2021 * DA [20]	Hong Kong	51	53.5	44 (86.3%)	Living with PwD (45) Unpaid (51) Family(51) -Spouses (6) 11.8%	NA	NA	NA	NA	GAD-7 CES-D PSS-10 ZBI	12 weeks	Significant increase in depression symptom scores; no changes in anxiety, and caregiver distress	Some
Giebel et al., 2021 [21]	United Kingdom	149	62 (13)	118 (79.7%)	NA Unpaid (149) Family or friend (149) -Spouses (NA)	72 (10)	14 (37.8)	AD (14) VAD (8) Others (15)	NA	PHQ-9 GAD-7	12 weeks	No significant changes in mental well-being	High
Hicks et al., 2022 [22]	United Kingdom	114	66.1 (13.81)	76 (69%)	Living with PwD (77) Unpaid (114) Family (114) -Spouses (NA)	79.5 (8.85)	79 (58%)	AD (65) DLB (6) VAD (16) Others(27)	Severe (5) Moderate (25) Mild (84)	C-DEMQQL carer	12 weeks	Significant decline in quality of life	Some
Manini et al., 2021 * CD [23]	Italy	94	64.4 (14.7)	64 68.1%	Living with PwD (77) Unpaid (89) Family (89) -Spouses (42)	83.2 (5.5)	67 (71.3%)	AD (78) DLB (3) VAD (3) FTD (2) Others(8)	Severe (28) Moderate (33) Mild (33)	Neuropsychiatric Inventory Caregiver Distress Scale	8 weeks	Significant, but overall modest increase in caregiver distress	High
Moretti et al., 2021 * DA [24]	Italy	221	NA	NA	Living with PwD (NA) Unpaid (221) Family (221) -Spouses (NA)	75.6 (6.6)	119 (54%)	VAD (221)	NA	RSS BDI HAM-A	8 weeks	Increase in depression, anxiety, and distress	High
Panerai at al., 2020 * CB [25]	Italy	128	57.5	94 (73.4%)	Living with PwD (NA) Unpaid (128) Family (128) -Spouses (57)	76	67 (52.3%)	AD (31) VAD (42) FTD (8) Others(47)	Severe (22) Moderate (47) Mild (59)	Caregiver Burden Inventory (CBI) NPI-Q	8 weeks	Increase in caregiver burden and distress	Very high
Perach et al., 2022 * A [26]	United Kingdom	114	66.1 (13.8)	76 (67%)	Living with PwD (77) Unpaid (NA) Family (107) -Spouses (63)	79.8 (8.9)	66 (58%)	NA	NA	National Statistics ONS4	32.8 weeks	- No significant changes in psychological wellbeing and anxiety	Some
Rajagopalan et., 2022 * DA [27]	India	66	46.18 (16.11)	18 (27.24)	Living with PwD (NA) Unpaid (66) Family (66) -Spouses (18)	67.48 (9.46)	33 (50.0%)	AD (20) VAD (9) FTD (14) Others(23)	Severe (13) Moderate (23) Mild (30)	NPI-CD DASS-21	21.4 weeks	No significant increase in depression, anxiety, and distress	High
Vernuccio et al., 2022 * CD [28]	Italy	100	NA	NA	Living with PwD (95) Unpaid (NA) Family (NA)	77.1	59 (59%)	AD (34) VAD (13) DLB (1) FTD (2) MCI (28) Others(22)	Severe (42) Mild (30)	NPI-CD	40 weeks (Between two evaluation time)	Caregiver’s distress increased	Some
Total	Asia:3 Europe:12	1702	60.40 (12.9)	65.7%	Living with PwD (72.9%) Unpaid (99.6%) Family (99.2%) -Spouses (52.2%)	76.36 (9.32)	52.0%	AD (36.4%) VAD (25.5%) MCI (5.4%)	Severe dementia (18.9%) Moderate (27.9%) Mild (53.2%)		23.15 weeks	- 5 studies: No changes in wellbeing. - 10 studies: Worsening psychological well-being	Some: 7 High: 6 Very High:2

Abbreviations: CB: Caregiver Burden; Caregiver Burden Inventory: CBI; CES-D: Center for Epidemiologic Studies Depression Scale; DASS-21: Depression Anxiety and Stress Scale; Hospital Anxiety and Depression Scale: GAD-7: Generalized Anxiety Disorder Scale; HADS; Resilience Scale for Adults: PHQ-9: Patient Health Questionnaire; PSQI: Pittsburgh Sleep Quality Index; ZBI: Zarit Burden Interview; Perceived Stress Scale-10 (PSS-10); HAM-A: Hamilton Anxiety Rating Scale; Beck’s Depression Inventory: BDI; RSS: Relative Stress Scale; NPI-CD, Neuropsychiatric Inventory Caregiver Distress; DASS-21, Depression, Anxiety and Stress Scales; Patient Health Questionnaire-9 (PHQ-9). NA: Not applicable. * = included in meta-analysis D: depression, A: anxiety, CB: caregiver’s burden, CD: Caregiver distress.

Two studies did not specify the type of dementia [20,26]. The prevalence of Alzheimer’s disease (AD), vascular dementia, and other types of dementia were 36.4%, 25.5%, and 32.7%, respectively, with 5.4% having MCI. In nine studies, dementia was reported as moderate (27.9%) or severe (18.9%): six studies did not define the severity. Almost all caregivers were unpaid family members (>90% were unpaid or were family) and were living with PwD (72.9%).

Multiple scales were used to describe the impact on caregivers’ psychological well-being: The Neuropsychiatric Inventory (NPI) (*n* = 5); Caregiver Burden Inventory (CBI) (*n* = 3); Generalized Anxiety Disorder Scale-7 (GAD-7) (*n* = 3); Depression Anxiety and Stress Scale (DASS) (*n* = 2); Zarit Burden Index (ZBI) (*n* = 2); c-Dementia Quality of Life Instrument (*n* = 2). One study used The Hospital Anxiety and Depression Scale (HADS), Patient Health Questionnaire-9 (PHQ-9), CarerQol-7D/CarerQol-VAS, National Statistics ONS4, and Center for Epidemiologic Studies Depression Scale (CED). The lockdown duration varied between 8 and 61 weeks (mean 23.15 weeks).

Across all studies (summarized in Table 1), there were no changes in psychological well-being in five studies [18,19,21,26,27] and worsening psychological well-being in ten studies.

### 3.3. Meta-Analysis (Figure 2)

There was high heterogeneity across studies for all outcomes (less for caregiver’s burden). The Egger’s test was marginally significant for a possible publication bias for studies of anxiety (*p* = 0.06), but the trim-and-fill analysis left the SMD unchanged. There was no publication bias for any other outcome.

**Figure 2:**
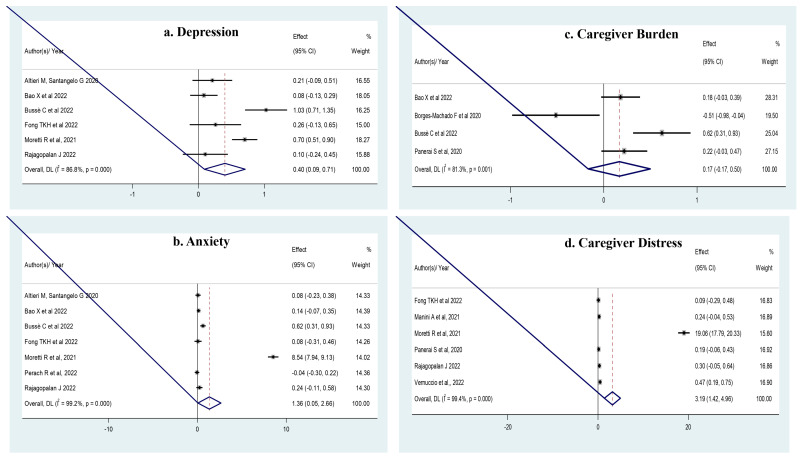
(**a**) There was a significant increase in **depression** (six studies [11,15,16,20,24,27], *n* = 1368; SMD = 0.401; 95% CI: 0.091 to 0.711; *p* = 0.01; *I*^2^ = 86.8%). (**b**). In **anxiety** (seven studies [11,15,16,20,24,26,27], *n* = 1569; and SMD = 1.357; 95% CI: 0.055–2.659; *I*^2^ = 99.2%). (**c**). In **caregiver’s burden** (four studies [11,16,17,25], *n* = 852; SMD = 0.34; 95% CI: 0.13 to 0.56; *p* = 0.001; *I*^2^ = 54.1%). (**d**). In **caregiver’s distress** (six studies [20,23,24,25,27,28], *n* = 1320; SMD = 3.190; 95% CI: 1.423–4.957; *p* < 0.0001; *I*^2^ = 99.4%).

### 3.4. Meta-Regression and Sensitivity Analyses

We pre-planned several meta-regression and sensitivity analyses, such as paid vs. unpaid caregivers, longitudinal data compared within the pandemic vs. before the pandemic, different outcome durations, dementia vs. MCI, early vs. late onset dementia, and the time without vs. with vaccination. However, the outcomes included less than ten studies, or the strata did not reach the minimum of four publications; thus, these analyses were not possible.

### 3.5. Risk of Bias

**Figure 3:**
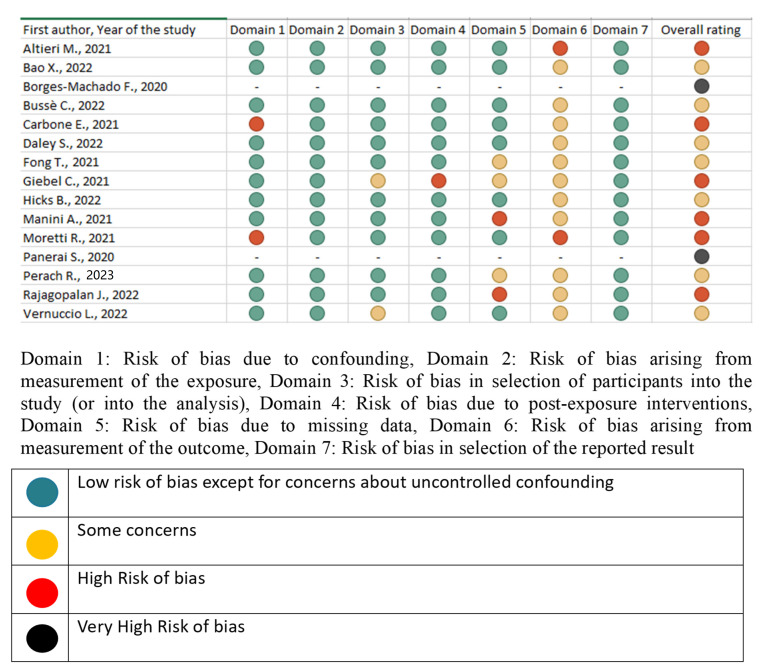
Evaluation of Risk of Bias [11,15,16,17,18,19,20,21,22,23,24,25,26,27,28].

The results of the risk of bias assessments are shown in Figure 3. Two studies were rated as very high risk of bias, six studies were rated as high risk of bias, and seven studies were rated as some concerns. Most of the studies had a high or very high level of bias (53.3%). The reasons for this might be due to the absence of those without dementia/MCI (i.e., a control group), not evaluating confounding factors, and the fact that caregivers’ psychological well-being was evaluated with a caregiver-based telephone interview.

## 4. Discussion

In this systematic review and meta-analysis of 15 studies including 1702 caregivers of PwD or MCI, caregiver psychological well-being parameters worsened during the pandemic for depression, anxiety, caregiver burden and caregiver distress. The studies had high heterogeneity, and many had a high risk of bias, particularly in outcome measurement, but the results suggest that the impact of COVID-19 on caregivers of PwD or MCI were important and may have implications for health and social care services in the future.

Depression, anxiety, caregiver burden and caregiver distress are often interrelated [20,29]; therefore, possible causes will be examined together. First, a previous study found that caregivers reported feeling prepared for typical caregiving responsibilities but felt less prepared for someone else to assume the role of primary caregiver. It is thus likely that, being unprepared as the COVID-19 pandemic emerged and took hold, both caregiver burden and distress increased [30]. Therefore, when one is diagnosed with dementia, caregivers, specifically unpaid and thus likely family members, should be informed and educated about how they can overcome the problems that may develop in those for whom they care (including extreme situations such as the COVID-19 pandemic, war, and disasters).

Globally, health systems were not prepared for the COVID-19 pandemic, subsequently causing caregivers to be adversely affected. During the COVID-19 pandemic, caregivers reported that they had difficulties in accessing medical services, which is not surprising considering that the COVID-19 pandemic led to the breakdown of health care systems worldwide and a decrease in the quality of health care due to overwhelmed wards or intensive care units. Amongst the most common problems described are the discontinuation of specialized medical care, difficulty accessing hospitals, or even appointments, and frequent COVID-19 antigen testing. Similarly, a recent European study described some of the frequent issues encountered: some hospitals had to reschedule non-urgent visits for safety measures, and people cancelled appointments because of the fear of infection [31]. Considering that the average age of PwD/MCI in our review is 76.36 years and the average age of caregivers is 60.4 years, it appears that caregivers may be concerned not only with dementia or COVID-19-related conditions, but also with other comorbid diseases and conditions associated with their own health problems. Indeed, in one study, caregivers stated that they restricted the use of health services for their own health problems during the pandemic, owing to fear of being infected with the virus during this process. This subsequently exacerbated existing health conditions among caregivers [32]. There is some evidence that telephone counseling can reduce depressive symptoms and meet the important needs of caregivers of PwD/MCI [33]. Also, online psychoeducational support and specific care guidelines can contribute to the well-being of PwD and caregivers [34]. Therefore, digital interventions for caregivers’ anxiety, depression, and burden should include video and online psychoeducational programs, telephone calls, and messages to reach those with poorer digital resources [29].

The COVID-19 pandemic and lockdown had a considerable impact on PwD as well as their caregivers: a systematic review and meta-analysis investigating the effects of the COVID-19 lockdown on neuropsychiatric symptoms (NPS) in PwD/MCI showed that there was an increase in the worsening of NPS (especially depression, anxiety, agitation, irritability, and apathy) in PwD/MCI during lockdown [7]. An increase in the NPS of PwD may explain why caregiver burden and distress increased during the pandemic. Indeed, it may be hypothesized that there is a bidirectional relationship between caregiver burden and NPS [35]. The inability to maintain physical activity and social interactions, which are non-pharmacological approaches recommended for NPS prevention, due to forced lockdown at home, and the closure of outpatient rehabilitation centers that provide services such as cognitive training, occupational therapy, and group activities, may have increased the risk for the development of NPS [8]. Additionally, the rapid cognitive deterioration in PwD during the pandemic, the challenges for these people to adapt to new living conditions, and the inability of people to continue their usual daily activities, may have led to the development of NPS [36]. It is clear that NPS are associated with negative caregiver outcomes. Indeed, depressive behaviors were the “most important symptoms” relative to caregiver burden, followed by agitation, aggression, sleep disturbances, and apathy [35]. Moreover, NPS may lead to a worsened relationship with the caregiver, and this altered relationship may lead people with NPS to be more irritable, or caregivers with a poorer relationship may perceive NPS as more severe, or as purposefully provocative behaviors. A study by Perren et al. found that the lower level of spousal caregivers’ well-being and insecure attachment style were associated with increased levels of NPS [37]. Interestingly, the inappropriate behavior of caregivers has been found to be associated with delusions; the impact of delusions on both the caregiver and PwD/MCI may be mitigated through pharmacological treatment. Moreover, previous studies have found that distressed caregivers tended to use emotion-oriented rather than problem-focus coping strategies, which may increase the person’s behavioral disturbances [38,39]. The caregiver’s irritation, anger, or impatience can lead to greater agitation in PwD/MCI [40]. One study also reported that caregiver-delivered cognitive stimulation therapy showed significant improvements in cognition, behavioral and psychiatric symptoms, and the quality of life of PwD [41]. Therefore, training caregivers, and combining pharmacological and behavioral and/or family interventions targeting NPS, may alleviate suffering for a PwD, but also mediate improved caregiver well-being. However, Bao et al. found that during the one-year COVID-19 lockdown, the change in PwD’s NPS was not associated with caregiver depression, anxiety, and burden [9]. Thus, it is clear that NPS is not the only factor influencing the psychological well-being of caregivers. Further, the lockdown period may be related to loneliness, social isolation, and reduced physical activity, caregiver anxiety, and depression [9]. Many studies suggest that social contact relieves anxiety and related disorders by activating a neural reward system, regulating the hypothalamic-pituitary-adrenal axis, and regulating and secreting neurotransmitters, including oxytocin and opioids [42]. Physical activity may promote mental health by decreasing anxiety and depression symptoms by downregulating TNF-α and other inflammation parameters [43]. Furthermore, countless hours spent caregiving could precipitate feelings of loneliness and intensify the distress levels of caregivers [5].

Owing to the potential pathways discussed above, the well-being of caregivers for older adults with dementia is especially important to examine during the COVID-19 lockdown. Moreover, depression, anxiety and distress has reduced among caregivers after COVID-19 restrictions (i.e., lockdown) ended [24]; this suggests the need for improved support of long-term lockdown strategies/policies, specifically for family caregivers. In the present review, we found that almost all caregivers were unpaid family members (>90% were unpaid or were family) and were living with PwD (72.9%). Family caregivers, in particular, reported higher anxiety, depression, fatigue, sleep disturbance, lower social participation, lower financial well-being, increased food insecurity, and increased financial worries [44]. Although the impact of dementia on the caregiver’s psychosocial well-being is persistent, increased cognitive impairment, decreased self-care ability, and caregiver burden, primarily anxiety, pressure, and distress in maintaining daily routines, also leads to a ‘caregiver burden’ during the pandemic [17,26].

The strength of our study is that it is the first compilation of the results of studies conducted under difficult pandemic conditions, with a rigorous search strategy, two independent reviewers at all stages, and a broad range of relevant outcomes. However, findings from the present study must be interpreted in light of its limitations. First, only a small number of studies could be included in the meta-analysis. It is noteworthy that studies focused on negative rather than positive outcomes, which may lead to confirmation bias. Second, nearly all caregivers were unpaid and there was a very small number of paid caregivers (0.4%). Therefore, we cannot generalize our results to paid caregivers. Third, the studies were clinically and statistically heterogeneous; this may be partly due to the different time periods between assessments, differences in dementia types and severity, and also evaluations of psychological well-being (by using different scales). Another limitation is that the data included in the studies only covers the first part of the pandemic. Additionally, the impacts of COVID-19 and restrictions may vary in different countries, which might be the cause of high heterogeneity and a high risk of bias.

## 5. Conclusions

There was an increase in depression, anxiety, distress, and caregiver burden in caregivers of PwD/MCI during the COVID-19 pandemic lockdown. Therefore, the COVID-19 pandemic had a negative effect on the psychological well-being of caregivers. There is a clear need to further explore the potential mechanisms relating to the negative outcomes, so that caregivers can be supported in any future similar scenarios, and supported with any ongoing symptoms as they continue to care for PwD/MCI, or deal with grief when their loved ones die.

## Data Availability

The data presented in this study are available if required.
